# Process Evaluation of the Tier 1 Program (Secondary 1 Curriculum) of the Project P.A.T.H.S.: Findings Based on the Full Implementation Phase

**DOI:** 10.1100/tsw.2008.4

**Published:** 2008-01-14

**Authors:** Daniel T. L. Shek, Hing Keung Ma, Rachel C. F. Sun, Daniel W. M. Lung

**Affiliations:** ^1^ Centre for Quality of Life, Hong Kong Institute of Asia-Pacific Studies, The Chinese University of Hong Kong, Hong Kong; ^2^ Kiang Wu Nursing College of Macau, Macau; ^3^ Social Welfare Practice and Research Centre, The Chinese University of Hong Kong, Hong Kong; ^4^ Department of Education Studies, Hong Kong Baptist University, Hong Kong

**Keywords:** process evaluation, observation, positive youth development program

## Abstract

To understand the implementation quality of the Tier 1 Program (Secondary 1 Curriculum) of the Project P.A.T.H.S. (Positive Adolescent Training through Holistic Social Programmes), observers carried out process evaluation in the form of systematic observations of 22 units in 14 randomly selected schools. Results showed that the overall level of program adherence was generally high (range: 45–100%, with an average of 86.3%). High implementation quality of the program in the areas of student interest, student participation and involvement, classroom control, use of interactive delivery method, use of strategies to enhance student motivation, use of positive and supportive feedbacks, instructors’ familiarity with the students, degree of achievement of the objectives, time management, lesson preparation, overall implementation quality, and success of implementation was also found. The present findings are consistent with those observations based on the experimental implementation phase, suggesting that the implementation quality of the Tier 1 Program (Secondary 1 Curriculum) was generally high.

### INTRODUCTION

Although different evaluation emphases exist in the evaluation literature in the international context[1,2,3], there are common evaluation frameworks and standards that are upheld by researchers in the mainstream scientific community. For example, the Centers for Disease Control and Prevention[4] suggested a comprehensive framework for program evaluation in public health. There are several steps in the proposed framework, including engaging the stakeholders, describing the program, focusing on the evaluation design, gathering credible evidence, justifying conclusions, and ensuring use and sharing lessons. Similar evaluation frameworks can be seen in the What Works Clearing House[5] that reviews and grades intervention programs in the context of education. Regarding evaluation standards, the Joint Committee on Standards for Education Evaluation[6] proposed several areas of evaluation criteria in different domains. In the above-mentioned models, the consequences of the program on the participants (i.e., outcome evaluation) and the quality of the implementation (i.e., process evaluation) are commonly regarded as important foci of evaluation frameworks.

According to Scheirer[7], process evaluation is “the use of empirical data to assess the delivery of programs… Process evaluation verifies what the program is, and whether or not it is delivered as intended to the targeted recipients and in the intended dosage” (p. 40). Although no evaluator would dispute against the importance of process evaluation, a review of the literature shows that research on process evaluation is not prevalent. With reference to the public health literature, Linnan and Steckler[8] commented that there is “a plethora of reports about interventions that have successful outcomes. A limited number of studies, however, disentangle the factors that ensure successful outcomes, characterize the failure to achieve success, or attempt to document the steps involved in achieving successful implementation of an intervention” (p. 1). In the context of evaluation of positive youth development programs, a survey of the literature shows that the related evaluation studies have focused primarily on objective outcome evaluation. This oversight is worrying, particularly in view of the fact that findings on program implementation quality are rarely reported by researchers[9,10,11].

There are several fatal consequences of overlooking the quality of the implementation of a program. First, the adoption of a “black box” approach (i.e., input-output analysis only) would make it difficult to understand the origin of the program success or failure. For example, the failure of a program may be due to the low program adherence, but not the program itself. Second, the lack of process evaluation would reduce the sensitivity of the program developers to look at the strengths and weaknesses of the programs developed. Third, process evaluation provides a vehicle for promoting communication and exchange between the program developers and program implementers. Finally, without process evaluation, program developers have to wait until the outcome data are collected if they wish to refine the program.

Against the above background, there are several arguments for conducting process evaluation[7]. First, process evaluation can tell the program developers whether a Type III error (i.e., existence or nonexistence of program effect because of occurrence of activities different from those intended by the program developers) has occurred. Second, fidelity in program implementation can be promoted by feedback collected in the implementation process. Third, process evaluation can help program developers to understand whether the intended targets receive the program. Fourth, process evaluation can help to identify factors that contribute to program success or failure. Finally, program developers can use process evaluation findings to understand how the developed program can be implemented successfully in human organizations and communities, which are always complex in nature.

In adolescent prevention programs, several process variables, such as opportunity for interaction, discretion in the coverage of program content, perceived effectiveness of previous prevention programs, perceived effectiveness of the program, support from school principal, nature of funding of the school, and adherence to the program, have been found to influence the program outcome[12]. Nation et al.[13] pointed out that there are many factors that determine the success of an adolescent prevention program, such as the use of a wide range of teaching methods that help the program participants to become aware of and understand problem behaviors and acquire the related skills. There are findings that show that the use of interactive methods and high peer interaction facilitate the positive effects of psychosocial intervention programs[14,15].

A survey of the literature shows that there are worrying trends and phenomena related to the development of adolescents in Hong Kong, such as mental health problems, abuse of psychotropic substances, adolescent suicide, school violence, and drop in family solidarity. As such, primary prevention programs that target specific adolescent developmental problems and positive youth development programs are called for[16]. However, research findings show that there are very few systematic and multiyear positive youth development programs in Hong Kong. Even if such programs exist, they commonly deal with isolated problems and issues in adolescent development (i.e., deficitsoriented programs) and they are relatively short term in nature. In addition, systematic and long-term evaluation of the available programs does not exist.

To promote holistic development among adolescents in Hong Kong, the Hong Kong Jockey Club Charities Trust has approved HK$400 million to launch a project entitled "P.A.T.H.S. to Adulthood: A Jockey Club Youth Enhancement Scheme". "P.A.T.H.S." denotes Positive Adolescent Training through Holistic Social Programmes. The Trust has invited academics of five universities in Hong Kong to form a research team, with The Chinese University of Hong Kong as the lead institution, to develop a multiyear universal positive youth development program to promote holistic adolescent development in Hong Kong. Besides developing the program, the research team also provides training for teachers and social workers who implement the program, and carries out longitudinal evaluation of the project.

There are two tiers of programs (Tier 1 and Tier 2) in this project. The Tier 1 Program is a universal positive youth development program where students in Secondary 1 to 3 will participate in the program, normally with 20 h of training in the school year at each grade. Because research findings suggest that roughly one-fifth of adolescents will need help of a deeper nature, the Tier 2 Program is generally be provided for at least one-fifth of the students who have greater psychosocial needs at each grade (i.e., selective program). To promote positive youth development, a total of 15 adolescent developmental constructs are covered in the project, particularly in the Tier 1 Program. These include: promotion of bonding, cultivation of resilience, promotion of social competence, promotion of emotional competence, promotion of cognitive competence, promotion of behavioral competence, promotion of moral competence, cultivation of self-determination, promotion of spirituality, development of self-efficacy, development of a clear and positive identity, promotion of beliefs in the future, provision of recognition for positive behavior, provision of opportunities for prosocial involvement, and fostering prosocial norms.

There are two implementation phases in this project – the experimental implementation phase and the full implementation phase. For the experimental implementation phase (January 2006 to August 2006), 52 secondary schools were invited to participate in the project with the objectives of accumulating experience in program implementation, and familiarizing front-line workers with the program design and philosophy. In the 2006/07 school year, the programs were implemented on a full scale at the Secondary 1 level. In the 2007/08 school year, the programs are implemented at the Secondary 1 and 2 levels. In the 2008/09 school year, the programs will be implemented at the Secondary 1, 2, and 3 levels.

There are several lines of evidence that support the effectiveness of the Tier 1 Program of P.A.T.H.S. First, evaluation findings based on a one-group pre/post-test design showed that there were positive changes in the program participants after joining the program[17]. Second, subjective outcome evaluation findings based on different studies, sources, and data types showed that the program participants and implementers had positive perceptions of the program, and they generally felt that the program was beneficial to the program participants[18,19,20,21]. Third, there are research findings that show that subjective outcome evaluation findings were related to objective outcome evaluation findings, with those who perceived higher benefits of the program showing greater positive changes on the different indicators of positive youth development[22]. Fourth, qualitative findings based on focus group interviews showed that the program participants enjoyed the program and they experienced positive changes in themselves[23]. Fifth, interim evaluation based on a random sample of 25 schools and three social work agencies showed that the respondents had positive perceptions of the program and its benefits to the program participants, although they also experienced difficulties and problems in the implementation[24]. Sixth, analyses of the students' weekly diaries showed that the students perceived that the program helped them in many areas and the participants generally enjoyed the program[25]. Finally, process evaluation based on systematic observations showed that the quality of implementation and program adherence were high[26].

Although the process evaluation based on the experimental implementation phase gave a favorable picture about the implementation quality of the Tier 1 Program, there was no guarantee that the implementation quality in the full implementation phase was acceptable. As such, process evaluation was carried out to examine the implementation quality of the Tier 1 Program (Secondary 1 Curriculum) based on a random sample of schools for the first year of the full implementation phase.

## METHODS

### Participants

Among the 207 schools that joined the Secondary 1 Program in the full implementation phase in 2006/07, there were 112 schools that adopted the full program (i.e., 20-h program involving 40 units) and 95 schools that adopted the core program (i.e., 10-h program involving 20 units). Among these schools, nine that adopted the full program and five that adopted the core program were randomly selected to conduct the observations. The characteristics of the schools that joined the process evaluation study can be seen in Table 1.

### Procedures

For each school joining the process evaluation study, systematic observations of one teaching unit or two teaching units were conducted. There were 22 units under observation, which covered 12 positive youth development constructs, including bonding, self-efficacy, prosocial norms, cognitive competence, emotional competence, moral competence, behavioral competence, resilience, self-determination, identity, spirituality, and beliefs in the future (see Table 1). The learning targets of these units can be seen in Table 2. The observers were six pairs of research assistants of the project who were registered social workers, with one social worker fixed in each pair. During the observations, each research assistant observed how the units were implemented and was required to complete a rating form covering four major areas, including basic information, integration with the school formal curriculum, program fidelity and adherence, and quality of program delivery (see Appendix 1) in an independent manner. For program fidelity and adherence, the observers rated the degree of adherence and recorded the time used to implement the unit. For the quality of program delivery, student interest, student participation and involvement, classroom control, use of interactive delivery method, use of strategies to enhance student motivation, use of positive and supportive feedbacks, instructors' familiarity with the students, opportunity for reflection, degree of achievement of the objectives, time management, quality of preparation, overall implementation quality, and success of implementation were rated. The research assistants did not have any discussion and they were "blind" to the ratings of their partner when they completed the rating forms.

## RESULTS

For every unit, the ratings of each item by the two independent observers were averaged. To obtain an overall picture, the ratings for each item across all units were again averaged. As the ratings of the observers were averaged, it was necessary to know whether the ratings were reliable. Based on the overall adherence ratings across the 22 units, Spearman correlation analyses showed that the ratings across the observers in the observed units (N = 22) were highly reliable (rho = 0.88, $p < 0.01$). The average overall adherence to the curriculum manuals was 86.3%, which was quite remarkable (Table 3). For those units where modifications had been made, the observers generally regarded the changes to be reasonable. Nevertheless, adherence for ID1.3 in School F was not high because one of the activities had overrun, which caused the remaining activities of this unit to be cancelled within the time limit.

The findings on the program implementation quality can be seen in Table 3. As the ratings were averaged across observers, it was necessary to know whether the ratings were reliable. Based on the mean overall ratings across the 22 units, Spearman correlation analyses showed that the ratings across the two observers in the observed units (N = 22) were moderately reliable (rho = 0.56, $p < 0.01$). Regarding the ratings for the quality of delivery, results in Table 3 revealed that the quality of implementation as assessed by the two observers was very high (over 5 on a 7-point rating scale). An examination of the different areas showed that the mean ratings were generally high, except that the opportunity for reflection was generally lower as compared to other dimensions of the program implementation quality. Also, implementation quality for School E and H was not very high.

**TABLE 1**
**Summary on the Characteristics of the Schools**

| School | A | B | C | D | E | F | G |
|---|---|---|---|---|---|---|---|
| | | | | ***Background Characteristics of the School*** | | | |
| Location (district) | Kwun Tong | Islands | Tuen Mun | Sham Shui Po | Eastern | Tai Po | Yau Tsim Mong |
| Finance mode | Aided | Aided | Aided | Aided | Aided | Aided | Aided |
| Sex composition | Coeducational | Coeducational | Coeducational | Coeducational | Coeducational | Coeducational | Coeducational |
| Religious background | Buddhism | Buddhism | Christianity | Christianity | Christianity | Taoism | Christianity |
| | | | | ***Context of Observation*** | | | |
| Choice of program | 10 h | 20 h | 10 h | 20 h | 20 h | 20 h | 20 h |
| Mode | 10 sessions (1 h per session) | 40 sessions (30 min per session) | 10 sessions (1 h per session) | 20 sessions (1 h per session) | 20 sessions (1 h per session) | 20 sessions (1 h per session) | 40 sessions (30 min per session) |
| Integration with school curriculum | Life Education | Class Teacher's Period | Class Teacher's Period | Life Education | Life Education | Religious Studies and Ethics | Life Education |
| No. of students in the class | 27 | 37 | 44 | 41 | 20 | 40 | 35 |
| Instructor(s) | 1 Teacher and 2 social workers | 1 Teacher | 1 Teacher | 1 Teacher and 1 social worker | 2 Teachers | 1 Teacher and 1 social worker | 2 Teachers |
| Duration of observation | 40 min | 35 min | 60 min | 70 min | 50 min | 80 min | 65 min |
| Unit(s) observed | PN 1.2 | SE 1.2 | BO 1.2 and 1.3 | SD 1.1 and 1.2 | BF 1.1 | ID 1.3 and 1.4 | SP 1.2 |
| **School** | **H** | **I** | **J** | **K** | **L** | **M** | **N** |
| | | | | ***Background Characteristics of the Schools*** | | | |
| Location (district) | Shatin | Kwun Tong | Wong Tai Sin | Sham Shui Po | Kwun Tong | Kwai Tsing | Shatin |
| Finance mode | Direct Subsidy | Aided | Government | Aided | Aided | Aided | Direct Subsidy |
| Sex composition | Coeducational | Girls | Boys | Coeducational | Girls | Coeducational | Coeducational |
| Religious background | N/A | Catholic | N/A | N/A | Christianity | N/A | Christianity |
| | | | | ***Context of Observation*** | | | |
| Choice of program | 10 h | 10 h | 20 h | 20 h | 10 h | 20 h | 20 h |
| Mode | 10 sessions (1 h per session) | 2 sessions (2.5 h per session) and 5 sessions (1 h per session) | 20 sessions (1 h per session) | 40 sessions (30 min per session) | 10 sessions (1 h per session) | 4 sessions (2.5 h per session) and 10 sessions (1 h per session) | 20 sessions (1 h per session) |
| Integration with school curriculum | Life Wide Learning | Liberal Studies | Life Education | Life Education | Liberal Studies | Liberal Studies | Life Education |
| No. of students in the class | 38 | 42 | 35 | 38 | 42 | 40 | 35 |
| Instructor(s) | 1 Teacher and 1 social worker | 2 Social workers | 2 Teachers and 1 social worker | 1 Teacher and 1 social worker | 2 Teachers | 2 Teachers | 1 Social worker |
| Duration of observation | 70 min | 60 min | 70 min | 30 min | 80 min | 40 min | 80 min |
| Unit(s) observed | EC 1.1 and 1.2 | MC 1.1 and 1.2 | BC 1.1 and 1.2 | SP 1.1 | CC 1.2 and MC 1.1 | RE 1.3 | SE 1.2 and 1.4 |

Results also showed that there was inter-relationship among the different dimensions of program implementation. Success of implementation (item 13) were positively correlated with student interest (item 1; rho = 0.84, $p < 0.01$), use of interactive delivery method (item 4; rho = 0.73, $p < 0.01$), use of strategies to enhance student motivation (item 5; rho = 0.77, $p < 0.01$), opportunity for reflection (item 8; rho = 0.66, $p < 0.01$), and time management (item 10; rho = 0.80, $p < 0.01$).

**TABLE 2**
**Summary on the Objectives of the Observed Units**

| School | Unit | Construct | Learning Target |
|---|---|---|---|
| A | PN 1.2 | Prosocial norms | To enhance students' understanding of the consequences of infringing local customs in a foreign society |
| B | SE 1.2 | Self-efficacy | To experience success; to identify past successes |
| C | BO 1.2 | Bonding | To learn about three major types of personality; to understand the effect of personality on interpersonal relationships |
| | BO 1.3 | Bonding | To identify the determinants for desirable and undesirable friends; to practice refusal principles and skills |
| D | SD 1.1 | Self-determination | To understand the meaning of autonomy; to comprehend the scope of autonomy and the prerequisites for exercising it |
| | SD 1.2 | Self-determination | To learn that one should consider and analyze others' opinions and the consequences of making a choice |
| E | BF 1.1 | Beliefs in the future | To understand the advantages and disadvantages of being optimistic and pessimistic |
| F | ID 1.3 | Clear and positive identity | To help students discover their qualities; to help students grasp how to discover their qualities by various methods and from different points of view |
| | ID 1.4 | Clear and positive identity | To understand that people reveal themselves to varying degrees; to understand that opening ourselves to others is a way of self-understanding and establishing our identities |
| G | SP 1.2 | Spirituality | To reflect on our relationship with nature; to appreciate the beauty of nature |
| H | EC 1.1 | Emotional competence | To talk about basic concepts of emotions; to articulate one's own emotional experiences |
| | EC 1.2 | Emotional competence | To distinguish between emotional states; to express and experience emotions in different ways |
| I | MC 1.1 | Moral competence | To understand the importance of cooperation in maintaining fairness; to understand the moral issues in maintaining fairness in our society |
| | MC 1.2 | Moral competence | To distinguish between proper and improper behaviors; to understand the importance of being self-disciplined and considerate in public places |
| J | BC 1.1 | Behavioral competence | To differentiate constructive criticism from destructive criticism; to understand the drawbacks of using destructive criticism; to learn skills for making constructive criticism |
| | BC 1.2 | Behavioral competence | To be able to make an apology (both verbally and nonverbally); to understand the importance of apologizing after doing something wrong in social interaction |
| K | SP 1.1 | Spirituality | To reflect on materialistic values and to weaken them |
| L | CC 1.2 | Cognitive competence | To apply rational thinking to the issue of "making Net friends"; to apply creative thinking to the issue of "making Net friends" |
| | MC 1.1 | Moral competence | To understand the importance of cooperation in maintaining fairness; to understand the moral issues in maintaining fairness in our society |
| M | RE 1.3 | Resilience | To identify five techniques to manage one's emotions in stressful situations; to identify positive thinking that can deal with negative emotions |
| N | SE 1.2 | Self-efficacy | To experience success; to identify past successes |
| | SE 1.4 | Self-efficacy | To set feasible goals; to understand that it is necessary to adjust goals to one's abilities |

**TABLE 3**
**Overall Ratings on Each Unit Observed in the Different Schools**

| School | A | B | C | | D | | E | F | | G | H | | I | | J | | K | L | | M | N | | Average |
|---|---|---|---|---|---|---|---|---|---|---|---|---|---|---|---|---|---|---|---|---|---|---|---|
| Unit observed | PN 1.2 | SE 1.2 | BO 1.2 | BO 1.3 | SD 1.1 | SD 1.2 | BF 1.1 | ID 1.3 | ID 1.4 | SP 1.2 | EC 1.1 | EC 1.2 | MC 1.1 | MC 1.2 | BC 1.1 | BC 1.2 | SP 1.1 | CC 1.2 | MC 1.1 | RE 1.3 | SE 1.2 | SE 1.4 | |
| Overall adherence (%) | 90 | 85 | 97.5 | 97.5 | 75 | 92.5 | 90 | 45 | 90 | 62.5 | 80 | 60 | 99.5 | 90 | 100 | 95 | 96.5 | 77.5 | 90 | 97.5 | 92.5 | 95 | 86.30 |
| **Ratings on a 7-point scale. The percentages of responses with ratings of 5 and above are in brackets.** | | | | | | | | | | | | | | | | | | | | | | | |
| **1. Student interest** | 5.5 (100) | 6 (100) | 6.5 (100) | 6.5 (100) | 6 (100) | 6 (100) | 3.5 (0) | 5.5 (100) | 5.5 (100) | 6 (100) | 5 (100) | 5 (100) | 6 (100) | 6.5 (100) | 5.5 (100) | 6 (100) | 5.5 (100) | 5.5 (100) | 5.5 (100) | 6 (100) | 5.5 (100) | 4.5 (50) | **5.61** |
| **2. Student participation and involvement** | 5.5 (100) | 6 (100) | 6 (100) | 6 (100) | 6 (100) | 6 (100) | 4.5 (50) | 5.5 (100) | 5.5 (100) | 6 (100) | 6 (100) | 5.5 (100) | 5.5 (100) | 6.5 (100) | 5.5 (100) | 5.5 (100) | 5.5 (100) | 5.5 (100) | 5.5 (100) | 6 (100) | 5 (100) | 4.5 (50) | **5.61** |
| **3. Classroom control** | 6 (100) | 6 (100) | 6.5 (100) | 6.5 (100) | 6.5 (100) | 6.5 (100) | 4.5 (50) | 5.5 (100) | 5.5 (100) | 5.5 (100) | 5 (100) | 5 (100) | 6.5 (100) | 6.5 (100) | 6.5 (100) | 5.5 (100) | 4.5 (50) | 5.5 (100) | 4.5 (50) | 6.5 (100) | 5 (100) | 4.5 (50) | **5.66** |
| **4. Interactive delivery method** | 4.5 (50) | 5.5 (100) | 6 (100) | 6 (100) | 6 (100) | 6 (100) | 5.5 (100) | 5 (100) | 5 (100) | 6 (100) | 3.5 (0) | 4.5 (50) | 5.5 (100) | 6 (100) | 5.5 (100) | 5.5 (100) | 5.5 (100) | 5 (50) | 5 (50) | 6.5 (100) | 5.5 (100) | 5 (50) | **5.39** |
| **5. Strategies to enhance student motivation** | 5.5 (100) | 6 (100) | 6 (100) | 6 (100) | 6 (100) | 6 (100) | 5 (50) | 5.5 (100) | 5.5 (100) | 5.5 (100) | 3.5 (0) | 4 (0) | 6.5 (100) | 6 (100) | 5.5 (100) | 5.5 (100) | 5 (50) | 4 (50) | 5 (100) | 5.5 (100) | 4.5 (50) | 4 (0) | **5.27** |
| **6. Use of positive and supportive feedbacks** | 6 (100) | 5.5 (100) | 6.5 (100) | 6.5 (100) | 6 (100) | 6 (100) | 5 (50) | 5.5 (100) | 5.5 (100) | 6 (100) | 4 (0) | 4 (0) | 6.5 (100) | 6 (100) | 6 (100) | 5 (50) | 4.5 (50) | 4.5 (50) | 5 (100) | 5 (50) | 4.5 (50) | 4 (0) | **5.34** |
| **7. Instructors' familiarity with the students** | 3.5 (0) | 6.5 (100) | 7 (100) | 7 (100) | 4.5 (50) | 4.5 (50) | 6 (100) | 6 (100) | 6 (100) | 7 (100) | 4.5 (50) | 5.5 (0) | 5 (50) | 4.5 (50) | 4.5 (50) | 5.5 (50) | 4.5 (50) | 5.5 (100) | 6 (100) | 5.5 (100) | 4.5 (50) | 4.5 (50) | **5.36** |
| **8. Opportunity for reflection** | 4 (0) | 5.5 (100) | 6.5 (100) | 6.5 (100) | 5.5 (100) | 5.5 (100) | 3 (0) | 5 (100) | 5.5 (100) | 5.5 (100) | 3.5 (0) | 3.5 (0) | 5.5 (100) | 5.5 (100) | 5 (100) | 5.5 (100) | 4.5 (50) | 5 (50) | 5.5 (100) | 4.5 (50) | 4 (50) | 4 (50) | **4.93** |
| **9. Degree of achievement of the objectives** | 6 (100) | 5.5 (100) | 6.5 (100) | 6.5 (100) | 6 (100) | 6 (100) | 5 (50) | 5 (100) | 5 (100) | 5 (100) | 4.5 (50) | 4.5 (50) | 6.5 (100) | 6.5 (100) | 6 (100) | 5.5 (100) | 5.5 (100) | 5 (50) | 5.5 (100) | 5.5 (100) | 5.5 (100) | 5 (50) | **5.55** |
| **10. Time management** | 6.5 (100) | 5 (100) | 6.5 (100) | 6.5 (100) | 5 (100) | 5 (100) | 4.5 (50) | 4 (0) | 4 (0) | 5 (50) | 5 (50) | 3.5 (0) | 6.5 (100) | 6.5 (100) | 5 (50) | 5 (50) | 5 (100) | 4 (50) | 4.5 (50) | 6 (100) | 5.5 (100) | 4.5 (50) | **5.14** |
| **11. Lesson preparation** | 5.5 (100) | 6.5 (100) | 6.5 (100) | 6.5 (100) | 6.5 (100) | 6.5 (100) | 4 (50) | 4 (0) | 4.5 (50) | 5.5 (100) | 5.5 (100) | 5.5 (100) | 6.5 (100) | 6.5 (100) | 6 (100) | 5 (100) | 5.5 (100) | 4.5 (50) | 5 (100) | 6.5 (100) | 6 (100) | 6 (100) | **5.66** |
| **12. Overall implementation quality** | 5.5 (100) | 6 (100) | 6.5 (100) | 6.5 (100) | 6 (100) | 6 (100) | 4 (0) | 5 (100) | 5 (100) | 5.5 (100) | 5 (100) | 4.5 (50) | 6.5 (100) | 6.5 (100) | 6 (100) | 5.5 (100) | 5 (100) | 4.5 (50) | 5.5 (100) | 5.5 (100) | 5.5 (100) | 4.5 (50) | **5.48** |
| **13. Success of implementation** | 5.5 (100) | 5.5 (100) | 6.5 (100) | 6.5 (100) | 6 (100) | 6 (100) | 4.5 (50) | 5 (100) | 5 (100) | 5.5 (100) | 5 (100) | 4.5 (50) | 6.5 (100) | 6 (100) | 6 (100) | 5.5 (100) | 5 (100) | 5 (50) | 5.5 (100) | 6.5 (100) | 5.5 (100) | 5 (50) | **5.55** |

### DISCUSSION

This paper attempts to examine the adherence and quality of implementation of the Tier 1 Program (Secondary 1 Curriculum) of the P.A.T.H.S. Project in the first year of the full implementation phase. With reference to the adherence of the program, results showed that the overall degree of adherence to the teaching units assessed by the two observers was on the high side. This observation is generally consistent with the previous findings[26], which showed that the mean program adherence was 84.5%. In short, the findings suggested that the need for modifying the units in the implementation process was not high. These findings would dispute the common myth that curricula-based positive youth development programs cannot be easily used and major modifications must be made for different adolescent populations.

The second major conclusion of the study is that the different aspects of the program delivery were perceived to be very positive. These aspects include (a) students' interest and involvement (item 1 and 2), (b) management and teaching strategies used by the instructors (items 3, 4, 5, 6, and 10), (c) instructors' relationship with the students and effort (item 7 and 11). In addition, the two observers perceived that the objectives of the units implemented could be achieved (item 9) and the overall quality of implementation was high (item 12). Most important of all, the implementation was regarded as successful by the observers (item 13). Nevertheless, the degree of reflection (item 8) was the lowest among the items. There are two possible explanations. First, the content of the units was too packed. Second, teaching style in Hong Kong is basically didactic in nature and does not encourage such kind of activity. Since reflection is an invaluable part of the learning process that encourages students to assess their growth, strengths, or weaknesses, and to apply the things they learned in their daily life, it should be addressed in the training provided to the instructors before they implement the program.

On the other hand, there were two schools where the scores on curriculum delivery were not good. Based on the observations of the observers, several factors may contribute to this situation: few opportunities for student reflection in the units observed in these schools, as well as low student interest in the unit BF.1.1, the lack of interactive delivery methods and motivating strategies used in the unit EC1.1, and poor time management in the unit EC1.2. Of course, unidentified factors contributing to these poor delivery methods are worth further exploration in future study.

There are several limitations of the study. First, because of manpower constraints, only 14 schools were randomly selected to participate in this study. Although the number of schools participating in the study can be regarded as respectable, it would be desirable to include more schools with different characteristics to participate in the study. Second, although the inter-rater reliability on the adherence was high, the reliability of ratings on the other aspects of the implementation process was only moderate. This may probably be due to use of different observers in different pairs. Third, besides adherence and the quality of implementation, process evaluation with reference to other dimensions, such as context of the implementation and the involvement of other stakeholders[27], would help the program developers to further understand the quality of the program implementation process. With reference to the comment of Linnan and Steckler[8] that "a number of gaps in current knowledge about process evaluation must be addressed if the field is to move forward" (p. 8), it is suggested that future studies should refine the concept of process, and the related assessment and interpretation methods.

Finally, consistent with the intrinsic problem of all observation studies where time sampling is involved, one needs to be conscious of the degree of generalizability of the present findings to other temporal and spatial contexts. One possible confounding effect is that the students may become more cooperative when there are visitors and outside observers. In addition, it is also possible that the workers might be more motivated to teach well when being observed. Of course, the use of ethnographic strategies with prolonged engagement and observations would be helpful. Despite these limitations and in conjunction with the previous research findings[23,26], the existing research findings suggest that the quality of implementation of the Tier 1 Program was high and the program was helpful to the program participants.

### ACKNOWLEDGMENTS

The preparation for this paper and the Project P.A.T.H.S. were financially supported by the Hong Kong Jockey Club Charities Trust.

## APPENDIX 1

#### P.A.T.H.S. to Adulthood: A Jockey Club Youth Enhancement Scheme

#### Tier 1 Program – Observation Form

### A. Basic Information

| | | | |
|---|---|---|---|
| (* Please copy this "☑" sign to the appropriate box.) | | | |
| Name of School: | | | |
| Form: ☐1 / ☐2 / ☐3 * Class: | | | |
| Number of Students: | Male: | Female: | |
| Number of Instructors: | Teacher(s): | Social Worker(s): | Others: |
| Sex of Instructors: | Male: | Female: | |
| Date of Observation: | | | |
| Duration of the Class Period: | | | |

### B. Integration with School's Formal Curriculum

| |
|---|
| (* Please copy this "☑" sign to the appropriate box.) |
| ☐ Incorporated into the formal curriculum (e.g., life education, civic education, liberal studies, etc.). Please specify the subject: ____________________<br>☐ Outside formal curriculum (e.g., after school, holiday, teachers' period, etc.) Please specify:<br><br>☐ Others (Please specify: ________________________) |
| Is the instructor the Form Teacher of the Class?<br>☐ Yes ☐ No |

### C. Program Fidelity and Adherence

The Unit implemented:

Instructions:

1. Please fill in all the names of the activities and their expected duration in chronological order as specified in the curriculum manual.
2. Please tick ✓'none' if the activity was not carried out at all;
   please tick ✓ 'all' if the activity was carried out with strict or high degree of adherence to the planned curriculum;
   please tick ✓ 'part' if the activity was modified, and please specify the modifications, for instance: alteration of teaching strategies, omission of key points or role plays, discussions, etc.

| **Activity** | | **Adherence to Planned Curriculum** | | | **Original Scheduled Time (mins)** | **Actual Time (mins)** |
|---|---|---|---|---|---|---|
| | | **None** | **Part (Estimated %) (Specify Modifications)** | **All** | | |
| 1. | | | | | | |
| 2. | | | | | | |
| 3. | | | | | | |
| 4. | | | | | | |
| 5. | | | | | | |
| 6. | | | | | | |
| 7. | | | | | | |

Overall speaking, the estimated degree of adherence to the planned curriculum is _____%.

### D. Assessment of Curriculum Delivery

1. STUDENT INTEREST: How interested were the students in this unit?

| □ 1 | □ 2 | □ 3 | □ 4 | □ 5 | □ 6 | □ 7 |
|---|---|---|---|---|---|---|
| None or very few were interested | | | Half were interested | | | All or nearly all were interested |

2. STUDENT PARTICIPATION AND INVOLVEMENT: To what extent did the students participate in class activities?

| □ 1 | □ 2 | □ 3 | □ 4 | □ 5 | □ 6 | □ 7 |
|---|---|---|---|---|---|---|
| None or very few participated | | | Half participated | | | All or nearly all actively participated |

3. CLASSROOM CONTROL: To what extent was the class controlled?

| □ 1 | □ 2 | □ 3 | □ 4 | □ 5 | □ 6 | □ 7 |
|---|---|---|---|---|---|---|
| Very poorly controlled | | | In between | | | Very well controlled |

4. INTERACTIVE DELIVERY METHOD: How interactive was the delivery method?

| □ 1 | □ 2 | □ 3 | □ 4 | □ 5 | □ 6 | □ 7 |
|---|---|---|---|---|---|---|
| Not interactive at all | | | Half interactive | | | Very interactive all the time |

5. STRATEGIES TO ENHANCE STUDENT MOTIVATION: To what extent were motivating strategies used to motivate the students?

| □ 1 | □ 2 | □ 3 | □ 4 | □ 5 | □ 6 | □ 7 |
|---|---|---|---|---|---|---|
| No motivating strategies at all | | | Half the time | | | Motivating strategies all the time |

6. USE OF POSITIVE AND SUPPORTIVE FEEDBACKS: How often were positive and supportive feedbacks elicited from the students?

| □ 1 | □ 2 | □ 3 | □ 4 | □ 5 | □ 6 | □ 7 |
|---|---|---|---|---|---|---|
| Not at all | | | Half the time | | | All or nearly all the time |

7. INSTRUCTORS' FAMILIARITY WITH THE STUDENTS (have to ask the instructors): To what extent did the instructor know the students?

| □ 1 | □ 2 | □ 3 | □ 4 | □ 5 | □ 6 | □ 7 |
|---|---|---|---|---|---|---|
| Not at all | | | Average | | | Very well |

| | | | | | | |
|---|---|---|---|---|---|---|
| 8. OPPORTUNITY FOR REFLECTION: To what extent was reflection encouraged? | | | | | | |
| □ 1 | □ 2 | □ 3 | □ 4 | □ 5 | □ 6 | □ 7 |
| Not at all | | | Half the time | | | All or nearly all the time |
| 9. EVALUATION OF THE DEGREE OF ACHIEVEMENT OF THE OBJECTIVES: To what extent were the objectives achieved? | | | | | | |
| □ 1 | □ 2 | □ 3 | □ 4 | □ 5 | □ 6 | □ 7 |
| Not achieved at all | | | In between | | | All or nearly achieved |
| 10. TIME MANAGEMENT: How well was the time managed? | | | | | | |
| □ 1 | □ 2 | □ 3 | □ 4 | □ 5 | □ 6 | □ 7 |
| Very poorly managed | | | In between | | | Very well managed |
| 11. LESSON PREPARATION: How well was the lesson prepared? | | | | | | |
| □ 1 | □ 2 | □ 3 | □ 4 | □ 5 | □ 6 | □ 7 |
| Poorly prepared | | | In between | | | Very well prepared |
| 12. OVERALL IMPLEMENTATION QUALITY: Overall speaking, do you think the quality of implementation of this unit is high? | | | | | | |
| □ 1 | □ 2 | □ 3 | □ 4 | □ 5 | □ 6 | □ 7 |
| Very low | | | Average | | | Very high |
| 13. SUCCESS OF IMPLEMENTATION: Overall speaking, do you think the implementation of the program was successful? | | | | | | |
| □ 1 | □ 2 | □ 3 | □ 4 | □ 5 | □ 6 | □ 7 |
| Very unsuccessful | | | Average | | | Very successful |

**E. Observer’s Feelings Towards the Lesson (open-ended question).**

**F. Other Comments/Observations (open-ended question).**

**G. Comments Made by the Instructor(s), If Any (open-ended question).**
